# Drought stress identification of tomato plant using multi-features of hyperspectral imaging and subsample fusion

**DOI:** 10.3389/fpls.2023.1073530

**Published:** 2023-02-28

**Authors:** Shizhuang Weng, Junjie Ma, Wentao Tao, Yujian Tan, Meijing Pan, Zixi Zhang, Linsheng Huang, Ling Zheng, Jinling Zhao

**Affiliations:** National Engineering Research Center for Agro-Ecological Big Data Analysis & Application, Anhui University, Hefei, China

**Keywords:** hyperspectral imaging, drought stress, tomato, multi-features, subsample fusion

## Abstract

Drought stress (DS) is one of the most frequently occurring stresses in tomato plants. Detecting tomato plant DS is vital for optimizing irrigation and improving fruit quality. In this study, a DS identification method using the multi-features of hyperspectral imaging (HSI) and subsample fusion was proposed. First, the HSI images were measured under imaging condition with supplemental blue lights, and the reflectance spectra were extracted from the HSI images of young and mature leaves at different DS levels (well-watered, reduced-watered, and deficient-watered treatment). The effective wavelengths (EWs) were screened by the genetic algorithm. Second, the reference image was determined by ReliefF, and the first four reflectance images of EWs that are weakly correlated with the reference image and mutually irrelevant were obtained using Pearson’s correlation analysis. The reflectance image set (RIS) was determined by evaluating the superposition effect of reflectance images on identification. The spectra of EWs and the image features extracted from the RIS by LeNet-5 were adopted to construct DS identification models based on support vector machine (SVM), random forest, and dense convolutional network. Third, the subsample fusion integrating the spectra and image features of young and mature leaves was used to improve the identification further. The results showed that supplemental blue lights can effectively remove the high-frequency noise and obtain high-quality HSI images. The positive effect of the combination of spectra of EWs and image features for DS identification proved that RIS contains feature information pointing to DS. Global optimal classification performance was achieved by SVM and subsample fusion, with a classification accuracy of 95.90% and 95.78% for calibration and prediction sets, respectively. Overall, the proposed method can provide an accurate and reliable analysis for tomato plant DS and is hoped to be applied to other crop stresses

## 1 Introduction

Tomato (*Solanum lycopersicum* L.) is a popular and important vegetable crop cultivated in more than 100 countries and regions (Xia et al., 2021). The tomato fruit possesses antiaging and cancer-preventing effects and is also valuable for human health as it contains natural antioxidants, such as lycopene, carotene, and vitamins, as well as organic acids, such as malic acid and citric acid (Eid et al., 2020; Mukhtar et al., 2020). During cultivation, tomato is inevitably subject to many biotic and abiotic stresses. Drought stress (DS) is the main factor affecting its growth and development. The rational utilization of water resources is one of the topics of global universal concern. How to identify DS degree accurately and optimize irrigation reasonably must be explored for the sustainable development of agriculture. Different molecular, biochemical, physiological, morphological, and ecological traits of plants are impaired under DS conditions (Seleiman et al., 2021), resulting in wilted leaves, small stem diameter, and reduced photosynthetic efficiency. Furthermore, DS can affect the concentration of nutrients, such as sugars, acids, and proteins, in tomato fruit and lead to a decline in yield and quality (Chen et al., 2014; Hao et al., 2019). The detection of tomato plant DS can assist in providing timely irrigation, ensure normal plant growth, improve fruit quality, and reduce economic loss (Moharana and Dutta, 2019).

Plants rely on leaves for photosynthesis and respiration to provide energy for themselves and exchange gases with the outside world (Flexas et al., 2006; Haworth et al., 2018; Rad et al., 2022). The leaf effectively summarizes the stress-driven perturbations of the plant’s physiological status (Melandri et al., 2021). Therefore, plant DS is usually characterized by the appearance, temperature, and optical properties of leaves. Visual analysis, canopy temperature, thermal imaging, machine vision, and spectroscopic techniques are commonly used to analyze the DS degree of plants. Visual analysis, which relies on professional and experienced inspectors, is convenient and nondestructive but susceptible to subjective interference (Weng et al., 2021). Canopy temperature and thermal imaging can quantify the complex relationship between temperature and stress degree without needing physical contact, but they are affected by the aliasing of plants and soil background information (Ni et al., 2015; Han et al., 2016). The low cost, noncontact, and rapid acquisition of the leaf’s external features are the major advantages of machine vision, however, the lack of information about the internal composition and structure of the leaf limits its identification accuracy (Taghizadeh et al., 2011; Pandey et al., 2017). In recent years, spectroscopic techniques, such as near-infrared spectroscopy and reflectance spectroscopy, have been widely used in plant DS assessment because of their simplicity, speediness, and zero reagent consumption, these techniques provide information on the stretching vibrations of hydrogen-containing functional groups, such as C—H, N—H, S—H, and O—H (Steidle Neto et al., 2017; Li P, et al., 2020; Das et al., 2021; Raddi et al., 2022). Nevertheless, the techniques cannot precisely locate the leaf on the designated plant. They may also be influenced by other plants. The lack of spatial information reflecting the color, texture, shape, and position of the leaf also limits the improvement of accuracy.

As the combination of two sensor modalities, namely, imaging and spectroscopy, hyperspectral imaging (HSI) is a promising nondestructive detection method that can provide spatial and internal information, such as the composition and molecular structure of analytes (Mishra et al., 2019; Sun et al., 2021). HSI technology is widely used in analyzing DS in plants. Chen et al. established the machine learning model using HSI to monitor the drought degree of tea seedlings under DS (Chen et al., 2021). Zhou et al. tested the application of hyperspectral reflectance as a high-throughput phenotyping approach for the early identification of DS in citrus trees by conducting a greenhouse experiment (Zhou et al., 2021). HSI has also been used to explore the physiological processes of DS in plants, which is essential for selecting drought-tolerant genotypes and promoting breeding research (Asaari et al., 2019). HSI has an excellent performance in many analyses but may hardly obtain a good recognition effect using only spectral information. For instance, the nondestructive detection of healthy leaves and leaves infected with grapevine leafroll disease based on the spectra from HSI obtained a classification accuracy of 60.74% to 89.93% in the first four phenological stages (Gao et al., 2020).

Recent studies have attempted to combine spectra and image features to gain adequate information and improve the application effects of HSI (Wang et al., 2015; Ru et al., 2019). Compared with the accuracy of identifying yellow rust in wheat leaves using the spectra alone, the accuracy of identifying yellow rust in wheat leaves using spectra and texture features was increased by 7.3%(Guo et al., 2020). Combining spectra, texture features, and morphological features can improve the accuracy by 2% and 1.3% for the germ side and endosperm side, respectively (Yang et al., 2015). The texture and morphological features of images were extracted in previous studies through statistical analysis methods, such as gray level co-occurrence matrix (GLCM) and morphological parameter calculation, whereas these methods are complex, time-consuming, dependent on spatial scale, and subject to prior information (Sachar and Kumar, 2021). In recent years, deep learning has demonstrated its excellent feature extraction ability and has been widely used, especially in the imaging field (Yu et al., 2020).

The leaf state in a single growth stage can hardly represent an accurate expression of plant stress because of limited multidimensional and heterogeneous information. Borraz-Martínez et al. examined young and adult leaves at the spectral level and found differential pieces of information between them (Borraz-Martinez et al., 2019). Subsample fusion, the integration of information from leaves at various growth stages, was explored to determine tomato plant DS.

In addition, illumination conditions considerably influence the image quality in HSI experiments. As a stable and diffuse light source, the halogen lamp is often used as an illumination unit in HSI. Nevertheless, the available light amount of the halogen lamp is low in the visible region, resulting in HSI images with a poor signal-to-noise ratio (SNR). Mahlein et al. indicated that supplemental visible light can alleviate this problem (Mahlein et al., 2015).

**Figure 1:**
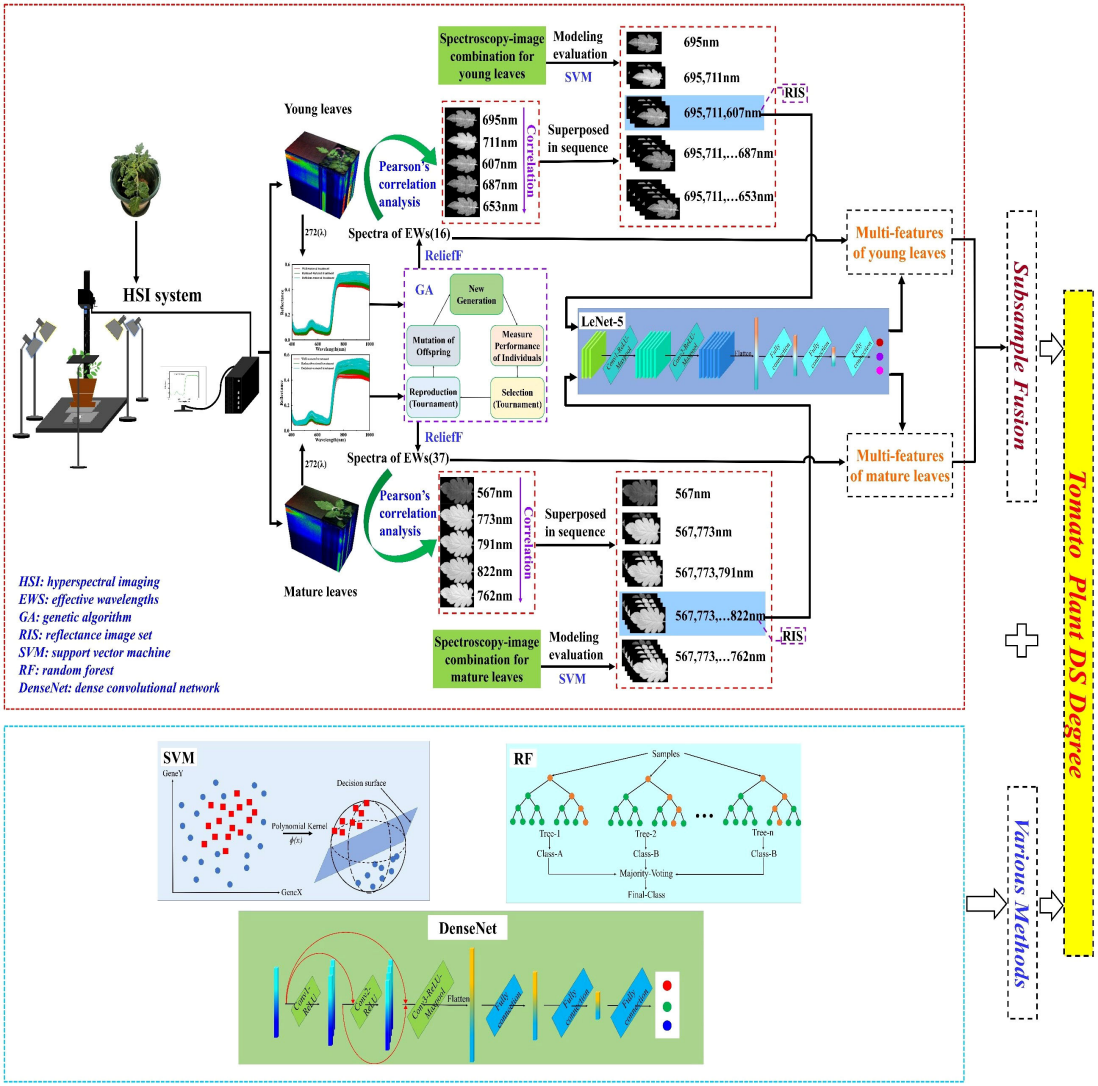
Flowchart of the determination of tomato plant DS degree.

The lack of light energy at 400–500 nm may be the main factor leading to high-frequency noise in the visible region. The spatial information may provide some help in accurately analyzing the plant DS. Moreover, the fusion of multiple types of leaf samples may enhance the judgment of plant physiological status. Herein, tomato plant DS was detected using multi-features of HSI and subsample fusion (**Figure 1**). In particular, the objectives of the present study were to (1) analyze the impact of blue lights on HSI image quality, (2) combine the spectra of the effective wavelengths (EWs) and image features extracted by LeNet-5 for DS analysis, (3) explore the effect of subsample fusion in DS analysis, and (4) develop the identification models of DS using dense convolutional network (DenseNet).

## 2 Materials and methods

### 2.1 Experimental design and irrigation treatments

The experiment was conducted in a greenhouse situated in the Laboratory of National Engineering Research Center for Agro-Ecological Big Data Analysis & Application, Anhui University, China. The red cherry tomato seedlings (50 days old) were purchased from the local market and transplanted into 18 pots (approximately 3 L each and one plant per pot) with ordinary soil on July 22, 2021. The available nitrogen, phosphorus, and potassium levels in the soil were determined by distillation method, spectrophotometric method, and flame photometric method, respectively. The fertilizer conversion ratio required to grow the tomato between the field and pot were calculated. Based on the calculation results, 1.603 g urea, 0.773 g phosphorus pentoxide, and 0.985 g potassium sulfate were added to the soil to promote tomato plant growth. The temperature and the relative humidity in the greenhouse were set at 24°C and 68%, respectively. The initial soil relative humidity (SRH) was maintained at 60%–80% by applying stored rainwater suitably. Appropriate and uniform light was also provided. After the plant roots were fixed, watering treatment was halted to reduce moisture. SRH was monitored daily by a temperature and humidity sensor. The samples of the three water treatments were obtained over time: (a) well-watered treatment: 60%–80% SRH; (b) reduced-watered treatment: 40%–60% SRH; (c) deficient-watered treatment: 20%–40% SRH.

### 2.2 HSI system and data acquisition

**Figure 2:**
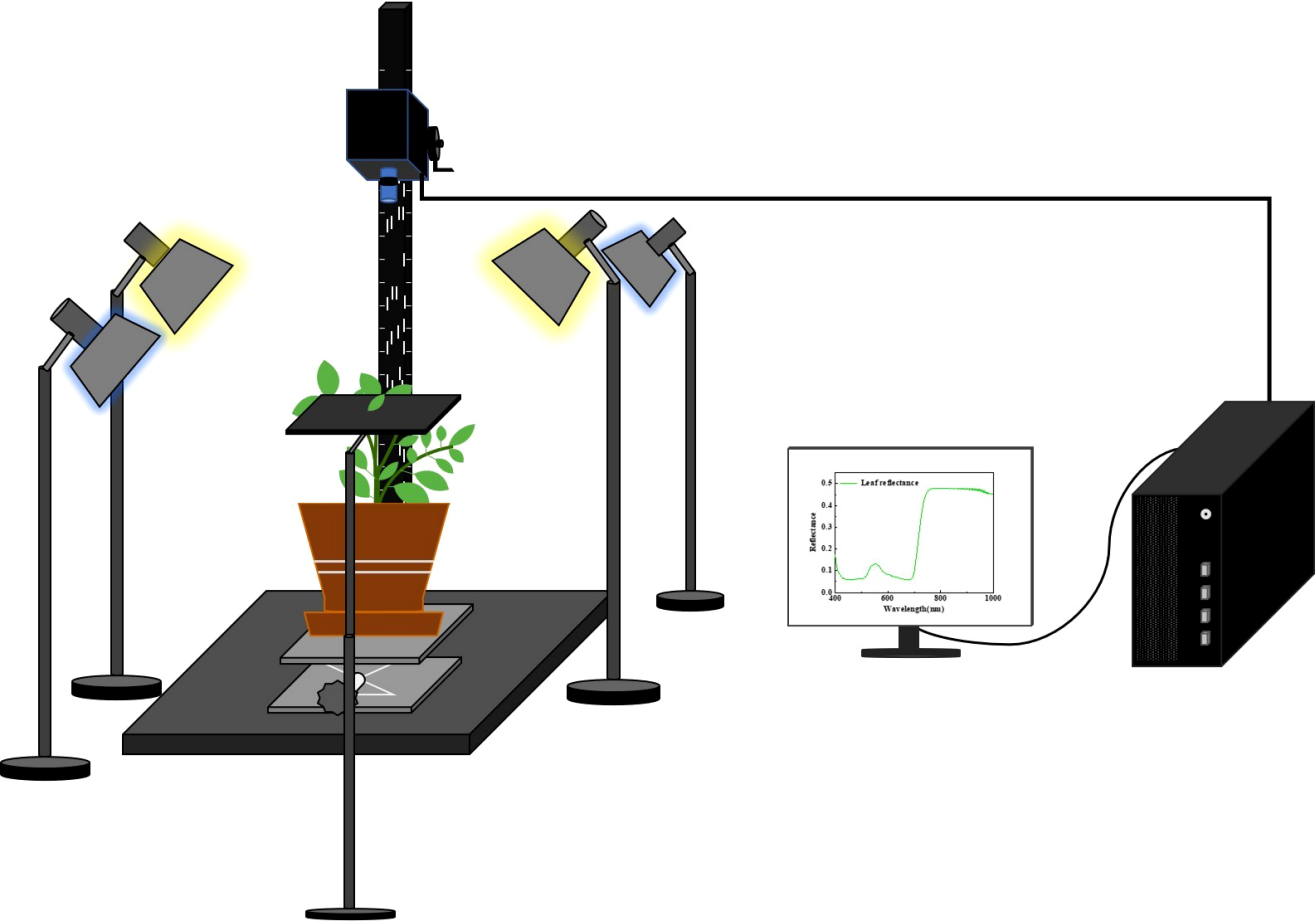
HSI system for tomato leaf data acquisition.


The HSI images of the leaves on tomato plants were collected using the HSI system in the visible/near-infrared range from 400 nm to 1000 nm (**Figures 2**; **S1**). The system consists of an indoor measuring platform with an area of 0.45 × 0.45 m^2^, a Headwall Nano-Hyperspec (Headwall Photonics Inc., Bolton, MA, USA) push-broom sensor that offers 272 spectral bands and 640 spatial pixels, a lighting unit with two halogen lamps (75 W) as the main radiation and two blue lights (3 W) as the auxiliary radiation, and a computing unit. Two halogen lamps and two blue lamps were placed on both sides and diagonal of the sample to ensure proper and uniform illumination. All lights were preheated for 30 min before imaging to reduce the influence of light intensity changes over time on the experiment (Ma et al., 2020). During data acquisition, the parameters of the HSI system were set as follows: exposure time, 60 ms; frame period, 65 ms; scanning speed, 0.543 deg/s. The distance between the lens and the sample was set at 30 cm by controlling the height of the lifting platform. For the image calibration, white and dark reference images were acquired by scanning a standard white board with 98% reflectance and covering the lens before collecting the HSI images of leaves. The correction formula is as follows:

(1) $$I_{c}=\frac{I_{r}-I_{d}}{I_{w}-I_{d}}$$

where *I*_c_ is the corrected image, *I*_r_ is the measured raw leaf image, and *I*_w_ a *I*_d_ are the white and dark reference images, respectively.

**Figure 3:**
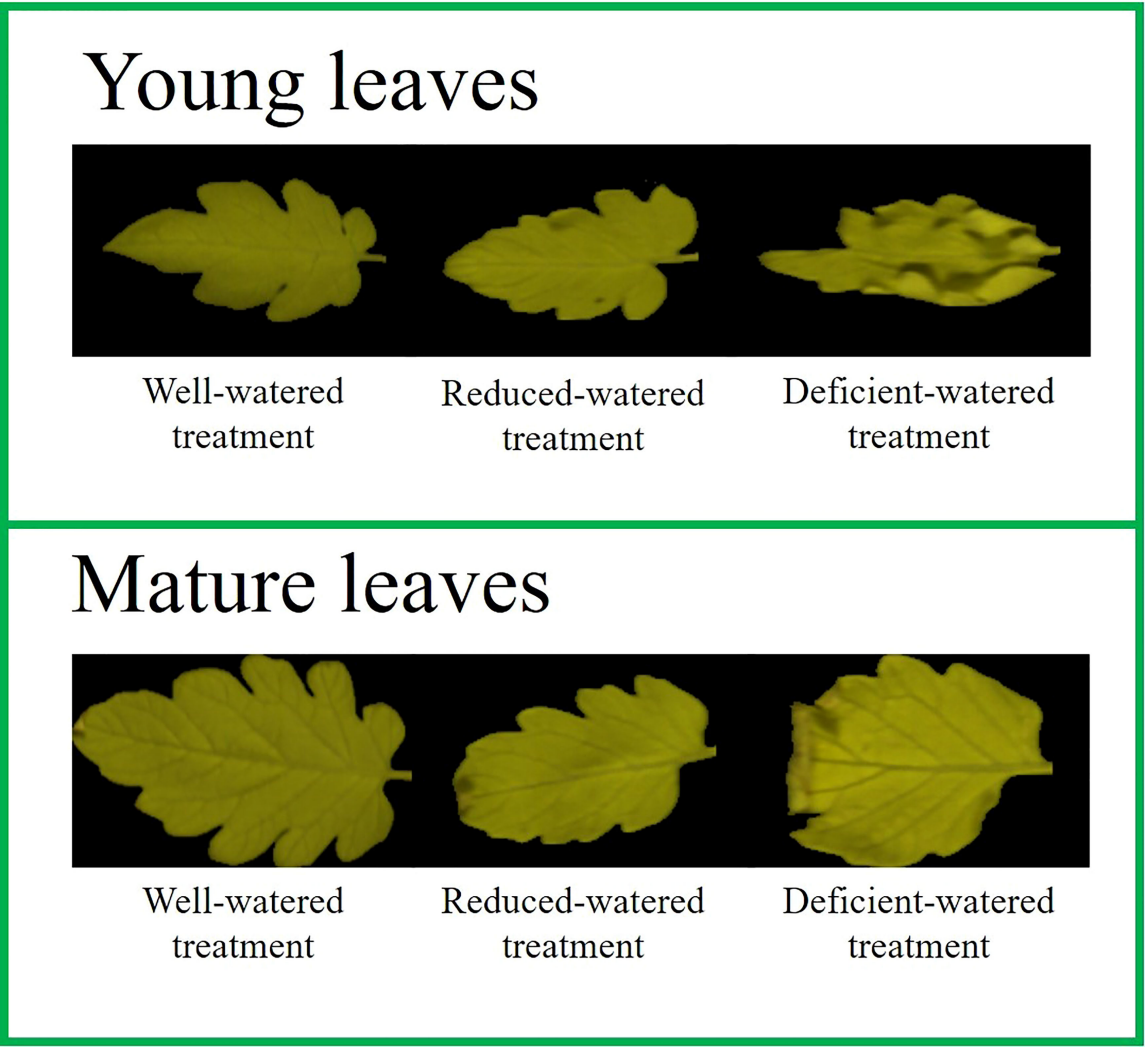
Color composite images of tomato leaves under different water treatments.

After the parameters of the HSI spectrometer were set, the first three leaves on the branch of the plant canopy were considered young leaves, and the last three leaves on the branch below the plant’s stem were mature leaves. Three young leaves (brightly colored with luster and toughness) and three mature leaves (dull colored without luster) were selected from each plant for HSI measurement. A total of 630 samples were obtained, including 315 images of young leaves and 315 images of mature leaves (**Figure 3**; **Table 1**).

**Table 1:** Sample distribution under different water treatments.

Datasets	Number of tomato leaf images		
	Well-watered	Reduced-watered	Deficient-watered
Young leaves	108	105	102
Mature leaves	108	105	102

### 2.3 Selection of EWs

After image correction, the region of interest (ROI) of the leaf was obtained by using the threshold segmentation method. The average value for all pixels within the ROI was calculated as the reflectance. The extracted reflectance spectra have high dimension and multicollinearity (Ng et al., 2019). A small number of variables can reduce the influence of noncorrelated variables, raise computational efficiency, and improve model performance. The genetic algorithm (GA), a variable selection method, was used to select EWs. The support vector machine (SVM) classifier was used as an evaluator. The algorithm parameters, such as initialized population, number of iterations, crossover probability, and variance probability, were set to 100, 400, 0.5, and 0.1, respectively. The fivefold crossover validation was employed to seek the global optimal solution in the descendants, and the mean value of cross-validation accuracy was used as the fitness function in this study. The EWs were determined by the following steps: (1) After the first application of GA, an accuracy value of the test set was obtained and used as the reference value. (2) GA was executed in a loop, and the number of the loop was set to 1000. If the run result was greater than the reference value, the result was designated as the new reference value. The loop was exited, and step (2) was repeated. (3) If GA was executed 1000 times continuously without obtaining a good result, the previously acquired feature subset would be considered the EWs. **Table S1** shows the selected EWs.

GA is an adaptive global probability search and optimization algorithm (Song et al., 2021) that utilizes selection, exchange, and mutation operations to retain the variables with high objective function values and delete the variables with low objective function values by continuous genetic iterations based on the biological evolution mechanism in nature. Thus, the optimal combination of variables was obtained.

### 2.4 Image features

The reflectance image of EW with the highest weight value given by ReliefF (Key et al., 2022) was regarded as the reference image. The reference image contains specific and crucial information. Other reflectance images with a weak correlation with the reference image can provide complementary information. Therefore, Pearson’s correlation analysis (Yang B, et al., 2021) was used to calculate the correlation between the reflectance images of EWs. Moreover, a threshold ranging from −0.3 to +0.3 was set. The reference image was placed in a defined container. Its correlation with the reflectance images of the remaining EWs (indexed sequentially) was analyzed until a reflectance image that met the threshold condition was found and added to the container. The correlation between the reflectance images of residual EWs and the reflectance images in the container was further calculated. A set of mutually unrelated reflectance images were obtained by threshold filtering. Then, the first four reflectance images with a weak correlation with the reference image were selected. Five reflectance images were superposed one by one according to the correlation ranking. The reflectance image set (RIS) was determined by analyzing the superposition effect of reflectance images. The RIS demonstrates high specificity, sensitivity, and convenience for further processing. Finally, the image features were extracted from RIS by LeNet-5. The relevant parameters are shown in **Table S2**.

Convolutional neural networks (CNNs), which use local connection and weight sharing to reduce the training parameters and computational complexity, can extract useful features quickly and accurately (Yang W, et al., 2021). CNNs are composed of convolution, pooling, and full connection layers. The convolution layer continuously learns the different characteristics of the input data. The pooling layer keeps the most important features while reducing the feature dimension to avoid overfitting. The full connection layer maps the resulting feature maps into a feature vector and generates a probability vector belonging to each class to achieve classification (Fazari et al., 2021; Weng et al., 2022). LeNet-5, a classical CNN, consists of two convolution layers, two pooling layers, two full connection layers, and one output layer (Priyadharshini et al., 2019). Image features were extracted by LeNet-5 without the final activation function.

### 2.5 Model construction

#### 2.5.1 Conventional machine learning methods

SVM is a supervised machine learning algorithm. It is often used to solve classification or regression problems owing to its excellent generalization ability. It maps data to a high-dimensional space through nonlinear transformation (defining an appropriate kernel function); then, it constructs the optimal separated hyperplane in the high-dimensional space to transform a nonlinear problem into a linear problem (Huang et al., 2019). Random forest (RF) can strongly prevent overfitting and resist noise, as it combines a large number of decision trees and averages the results of all the decision trees to determine the final classification type (Yang H, et al., 2021).

#### 2.5.2 Dense convolution neural networks

DenseNet, a mainstream learning method, was used to construct depth recognition models, enhancing feature transmission, encouraging feature reuse, and mitigating the gradient disappearance phenomenon (Li G, et al., 2020). It mainly comprises dense connecting blocks. Each layer in a dense block obtains additional inputs from all preceding layers and passes its feature maps to all subsequent layers, which can derive gradients directly from the loss function and the original input signal, leading to implicit deep supervision (Lawal, 2021). The spectra and image features were input as a 1D vector, and only one dense block was used in this study.

### 2.6 Performance evaluation

The HSI images of the tomato leaves that underwent three kinds of water treatments were divided into the calibration set and prediction set with a ratio of 7:3. In this study, the calibration set accuracy (ACC_C_), prediction set accuracy (ACC_P_), precision, recall, and F1-score of the prediction set, were used to evaluate model performance. All methods, including variable selection, image feature extraction, and model construction, were performed in Python 3.7.0. All programs were run on a computer with an Intel Core i7-3770 CPU, a main frequency of 3.40 GHz, and PyCharm software.

## 3 Results and discussion

### 3.1 Spectral analysis

**Figure 4:**
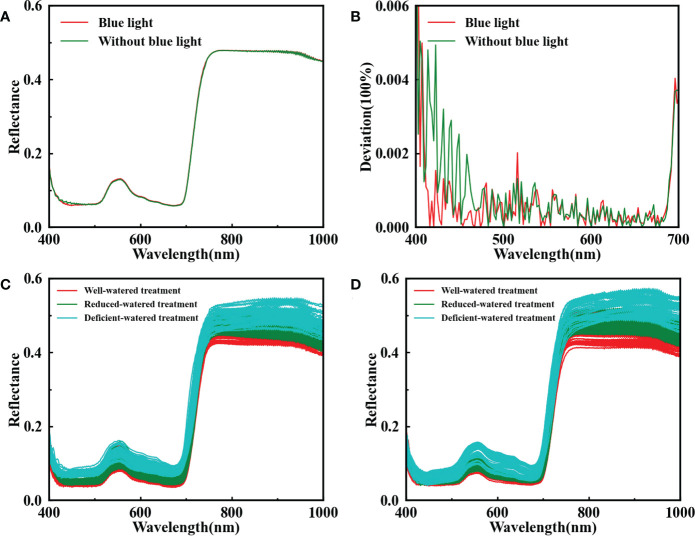
Reflectance spectra of the leaf with blue lights (red line) and without blue lights (green line) in the range of 400–1000 nm **(A)**; the deviation calculated by subtracting between the raw spectrum and the spectrum smoothed with Savitzky-Golay at 400–700 nm **(B)**; reflectance spectra for all available young leaves **(C)** and mature leaves **(D)** under different water treatments.

Blue lights (3 W) were used as supplementary lighting in this study. **Figure 4A** displays the spectral differences in the 400–1000 nm range, and the spectral curve without blue lights was affected by noise within the 400–500 nm range. The deviation plot (**Figure 4B**) shows considerable high-frequency noise in the spectrum without blue lights in the 400–500 nm range, indicating that the auxiliary lighting of blue lights can effectively improve image quality. It is also evidenced by the results of the parallel experiment in **Table S3**.

The reflectance spectra for all the available young and mature leaves of tomato plants under different water treatments with the supplementary illumination of blue lights are shown in **Figures 4C, D** respectively. The low reflectance in the 450–700 nm range was due to the strong absorption of chlorophylls A and B for blue and red lights. A convex peak was present at 560 nm, and a high degree of tomato plant DS indicated a high reflectance value. This finding suggests that DS would reduce the concentration of photosynthetic pigments and weaken the light absorption capacity. The red edge phenomenon near 730 nm and the reflectance at a high level within 780–1000 nm were controlled by the internal structure of the leaf. The tomato plants with a serious stress degree had great blue shift distance for spectra. Plant self-protection mechanisms, such as leaf dehydration, stomatal closure, and leaf curling, would be activated under DS. However, this physiological state feedback was delayed relative to the spectral response. In brief, spectral changes determine the feasibility of HSI in identifying tomato plant DS.

### 3.2 DS identification based on spectra

SVM, RF, and DenseNet were used to develop the recognition models of tomato plant DS based on the full spectra and the spectra of EWs of young and mature leaves (**Table 2**). The parameter settings of models are shown in **Table S4**. In terms of the full spectra of the young leaves, SVM, RF, and DenseNet had ACC_C_ values of 90.90%, 83.63%, and 95.45%, respectively, and ACC_P_ values of 87.36%,74.73%, and 87.36%, respectively. Similarly, SVM gained the optimal recognition result for mature leaves with ACC_C_ = 94.09% and ACC_P_ = 91.57%. The reason may be that the decision boundary of SVM was suited for the data distribution of tomato plant DS. With a powerful feature representation capability, DenseNet can accurately distinguish the DS levels of the tomato plant by combining low-level and deep features. RF performed slightly worse than the two other methods, it may be insensitive to tomato plant DS data.

**Table 2:** Classification results of tomato plant DS based on full spectra and spectra of EWs.

Data types	Categories	Methods/Accuracy (%)					
		SVM		RF		DenseNet	
		ACC_C_	ACC_P_	ACC_C_	ACC_P_	ACC_C_	ACC_P_
Full spectra	Young leaves	90.90	87.36	83.63	74.73	95.45	87.36
	Mature leaves	94.09	91.57	93.18	86.31	97.27	88.42
Spectra of EWs	Young leaves	95.45	90.52	81.81	73.68	90.00	88.42
	Mature leaves	95.45	92.63	93.63	86.31	98.63	89.47

EWs, effective wavelengths; ACC_C_, calibration set accuracy; ACC_P_, prediction set accuracy; SVM, support vector machine; RF, random forest; DenseNet, dense convolutional network.

**Figure 5:**
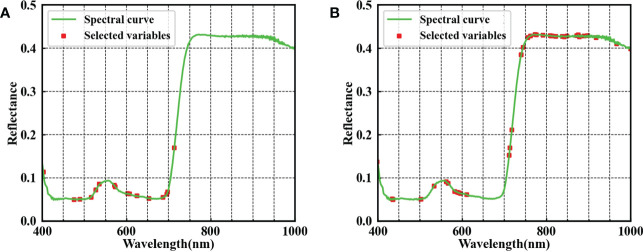
EWs selected by the GA of young **(A)** and mature **(B)** leaves.

Selecting the important variables beneficial to the learning algorithm can reduce the difficulty of the learning task and increase the interpretability of models. The spectra of EWs selected by GA were used to analyze tomato plant DS, and the ACC_P_ of the optimal SVM model improved by 3.16% for young leaves and 1.06% for mature leaves compared with the use of full spectra (**Table 2**). However, a slight deterioration in the identification result of RF was observed in young leaves because of a sharp decrease in the number of wavelengths. A total of 16 EWs for young leaves and 37 EWs for mature leaves were selected by GA, as shown in **Figure 5**. Specially, the EWs of young leaves all appear near the peak and valley in the visible region, meaning that young leaves are sensitive to changes in pigment concentration under DS. The EWs of mature leaves spread across the entire spectral range. Overall, the changes in the photosynthetic pigments and cell structure in the leaf were important indicators for evaluating tomato plant DS. The spectral information representing tomato plant DS differed between young and mature leaves. Therefore, the information on young and mature leaves can complement each other, probably facilitating DS analysis.

### 3.3 DS identification using spectra and image features

**Figure 6:**
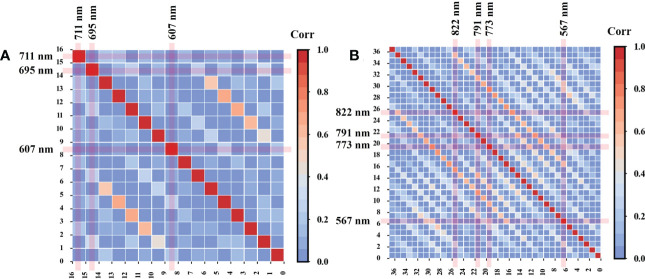
Pearson’s correlation between the reflectance images of EWs in young **(A)** and mature **(B)** leaves.

The direct use of leaf reflectance images with 272 wavelengths in HSI to identify tomato plant DS would consume a huge amount of time and reduce accuracy because of the high redundancy of HSI images. Knowing the correlation between reflectance images may be advantageous for extracting and synthesizing valid information and discarding useless information. Therefore, one young leaf sample and one mature leaf sample were randomly selected, and the correlation coefficient matrices of the EWs’ reflectance images were calculated using Pearson correlation analysis. The heat map indicated the weak and strong correlations between the reflectance images of EWs, Further screening of the RIS helped remove collinearity variables (**Figure 6**). The RIS was determined by modeling analysis based on SVM for the spectra of EWs and image features extracted by LeNet-5 from different reflectance image combinations obtained by increasing the number of images in a sequence according to the correlation ranking of the first four reflectance images with weak correlation with the reference image (**Table S5**). The final RIS included the images of 607, 695, and 711 nm for young leaves and 567, 773, 791, and 822 nm for mature leaves. As shown in **Figures 6A** and **6B**, the determined RIS was marked in the heat map. Any reflectance image in the RIS was weakly correlated with the other reflectance images, with correlation coefficients ranging from −0.3 to +0.3. Then, the image features extracted from RIS by LeNet-5 were combined with the spectra of EWs, called spectroscopy-image combination, to estimate the DS level (**Table 3**). The addition of new features resulted in different responses for the models. The accuracy of all models, except RF, was improved for young leaves, possibly because of the weak adaptability of RF to heterogeneous features and the presence of nonnegligible noise in image features. In regard to the mature leaves, the classification accuracy was improved by 1%–2% after the spectroscopy-image combination. The parameters of SVM, DenseNet, and RF are illustrated in **Table S6.** In general, the image features can replenish the missing spatial information, and spectroscopy-image combination offers an accurate stress analysis.

**Table 3:** Classification results of tomato plant DS based on spectroscopy-image combination.

Strategy	Categories	Methods/Accuracy (%)					
		SVM		RF		DenseNet	
		ACC_C_	ACC_P_	ACC_C_	ACC_P_	ACC_C_	ACC_P_
Spectra of EWs + image features	Young leaves	94.09	91.57	80.90	70.52	90.45	89.47
	Mature leaves	96.36	93.68	99.09	87.36	98.18	90.52

### 3.4 Effect of subsample fusion on DS analysis

**Figure 7:**
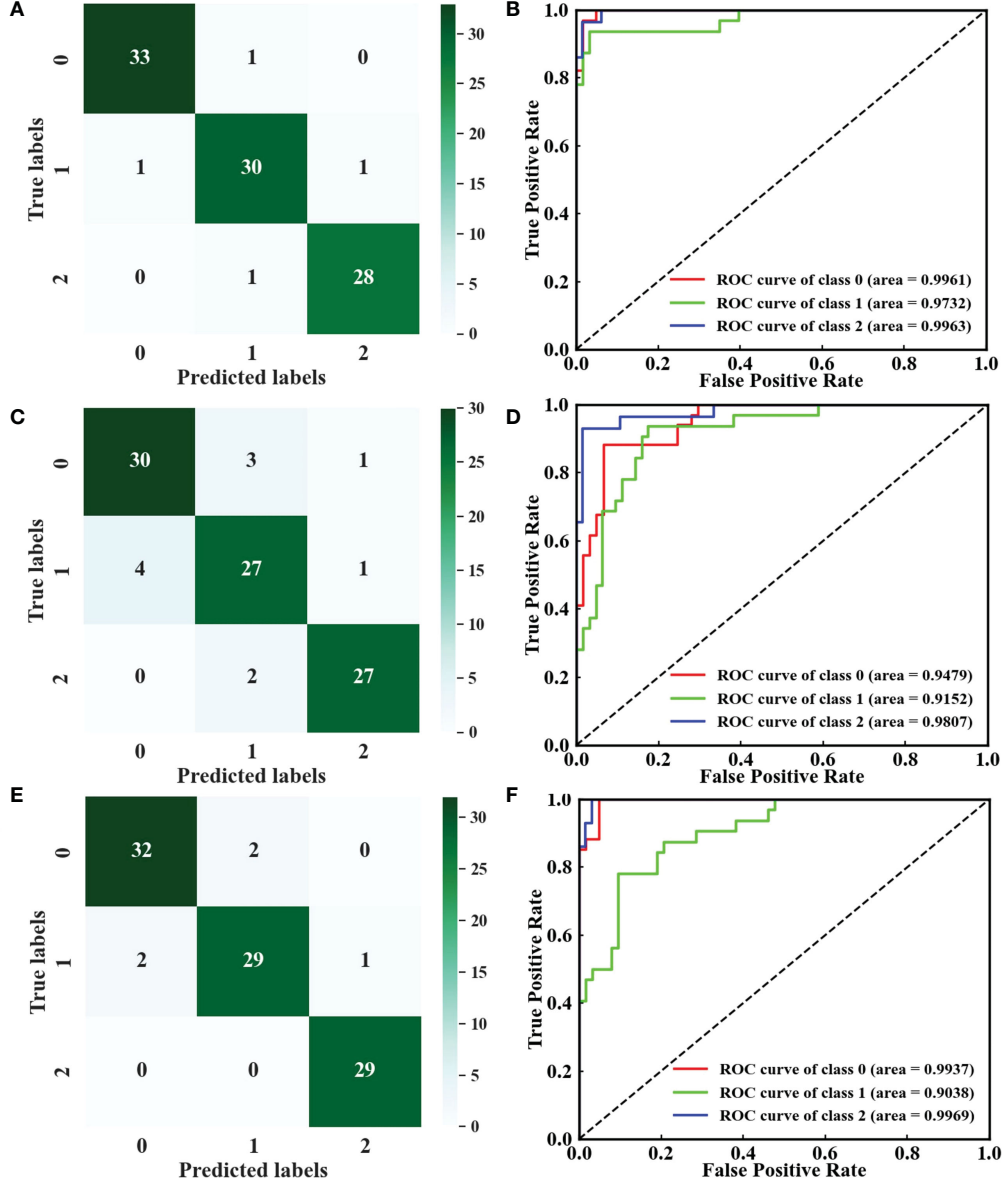
Confusion matrices and ROC curves of **(A, B)** SVM, **(C, D)** RF, and **(E, F)** DenseNet. Classes 0–2: well-watered, reduced-watered, and deficient-watered tomato plants, respectively.

The tomato leaves at different growth stages showed varied characteristics in color, composition, and appearance. The spectra and image features of young and mature leaves would provide the multilevel information of tomato plant DS. After subsample fusion (**Table 4**), a global optimal result of ACC_C_ = 95.90% and ACC_P_ = 95.78% was achieved by SVM. DenseNet also showed a strong identification ability of tomato plant DS. The detailed parameter settings of the models are shown in **Table S7**. The precision, recall, and F1-score values for the tomato plants with reduced-watered treatment were slightly lower than those in the two other water treatments. The reason is that reduced-watered treatment was in the intermediate state during the whole process of DS and easy to be misclassified into other classes. The confusion matrices of SVM, RF, and DenseNet (**Figures 7A, C, E**) showed that the tomato plants with reduced-watered treatment were easily misclassified as well-watered or deficient-watered tomato plants owing to inapparent differences in characteristics. Furthermore, the receiver operating characteristic (ROC) curves clearly showed the strong specificity and high sensitivity of SVM, followed by DenseNet and RF. The dispersed distributions of the ROC curves under the three water treatments indicated the presence of variances recognizing different classes (**Figures 7B, D, F**). Subsample fusion could augment the information difference of inter-class samples to promote further the effective identification of tomato plant DS. SVM performed well in identifying tomato plant DS with excellent accuracy and satisfactory robustness.

**Table 4:** Classification results of tomato plant DS based on subsample fusion.

Methods	Classes	Accuracy (%)	Prediction Set		
			Precision (%)	Recall (%)	F1-score (%)
SVM	Well-watered	ACC_C_=95.90 ACC_P_= 95.78	97.06	97.06	97.06
	Reduced-watered		93.75	93.75	93.75
	Deficient-watered		96.55	96.55	96.55
RF	Well-watered	ACC_C_= 96.81 ACC_P_= 88.42	88.24	88.24	88.24
	Reduced-watered		84.38	84.38	84.38
	Deficient-watered		93.10	9310	93.10
DenseNet	Well-watered	ACC_C_= 97.27 ACC_P_= 94.73	94.12	94.12	94.12
	Reduced-watered		93.55	90.62	92.06
	Deficient-watered		96.67	100.00	98.31

This study aims to provide a definite management decision for the rapid identification of tomato plant DS based on the multi-features of HSI and subsample fusion, which has significance in agricultural crop management and production practices (**Table 5**).

**Table 5:** Significance of results.

Points	Significance
Supplement blue light	Suppress high-frequency noise and improve imaging quality effectively
Variable selection	Reduce computational cost and optimize classification performance
Spectroscopy-image combination	Synthesize multidimensional (spectral and spatial) information and improve information utilization
Subsample fusion	Integrate heterogeneous information and enhance classification model recognition ability

### 3.5 Discussion

#### 3.5.1 Feasibility of HSI in plant DS

Traditional approaches for assessing plant DS include canopy temperature (Taghvaeian et al., 2014), chlorophyll fluorescence (Kautz et al., 2014), and thermal imaging (Jose Blaya-Ros et al., 2020). However, these measurements have low sensitivity and poor accuracy in practical applications, limiting their utility for rapid detection in the field. Furthermore, the concentration of substances in the leaves had already changed before DS caused changes in leaf traits. Recently, HSI has been widely used as a mainstream, rapid, and nondestructive measurement method in agriculture to obtain plant biological information reflecting metabolic changes (Zhao et al., 2020). Elvanidi et al. applied HSI to the detection of changes in the spectral reflectance of tomato plants under varying irrigation regimes to estimate plant water status under a controlled environment (Elvanidi et al., 2018), demonstrating the feasibility of HSI for nondestructive observation in tomato plant DS.

#### 3.5.2 Utilization of spatial information

However, the utilization of spectra alone cannot show the imaging advantage of HSI in many spectral technologies, and the lack of spatial information leads to unsatisfactory results. Spectroscopy-image combination can fully use the information in the HSI images and effectively avoid information loss. Few previous studies have discussed the spectroscopy-image combination to identify DS in tomato plants. The superiority of the spectroscopy-image combination in this study may be due to the fusion of internal and external attributes in tomato leaves. However, most studies only applied wavelength selection methods to gain RIS (Weng et al., 2021), ignoring the relevancy degree between reflectance images. Wavelength selection methods rely on the reflectance values obtained by averaging all the pixels of the reflectance image. Correlation analysis directly considers the global information of the image. RIS was screened by comprehensively considering the spectral attributes and correlations between the images in this study. Simultaneously, image features were generally extracted by image statistical methods, such as GLCM, local binary pattern, and color moment (Xie et al., 2015; Lu et al., 2019). Sachar et al. summarize some methods for leaf image feature extraction without mentioning deep neural networks (Sachar and Kumar, 2021). Feature extraction using deep neural networks was proved feasible by Yang et al. and Zheng et al. (Yang W, et al., 2021; Zheng et al., 2022). Thus, LeNet-5 was used to extract image features automatically using the flexible network structure, avoiding complex mathematical analysis. The classification accuracy values of the spectroscopy-image combination models were ACC_C_ = 94.09% and ACC_P_ = 91.57% for young leaves and ACC_C_ = 96.36% and ACC_P_ = 93.68% for mature leaves, which were better than those of the model based on the spectra or the image features alone (**Table S8**). During the DS analysis of the plant, the possible reasons for the poor results obtained by relying only on images to distinguish DS degree are the insignificant differences in the appearance of tomato leaves with different stress levels, the influence of noise, and the low spatial resolution of the HSI images. Combining spectral and spatial information would be an effective solution for the first reason. Then, the noise can be removed by suitable supplemental lighting or by developing algorithms. In addition, super-resolution image reconstruction techniques are considered for enhancing the spatial resolution and improving the sensitivity of DS response.

#### 3.5.3 Fusion strategy

The leaves at different growth stages of the same plant have different responses to DS. Other studies may point to the differences between young and mature leaves, but the data fusion approach for promoting DS analysis has rarely been applied. Subsample fusion, integrating the spectra and image features of young and mature leaves, can provide the differential information of multiple types of samples and reduce generalization error. The classification accuracy of the subsample fusion model was further improved, with ACC_C_ = 95.90% and ACC_P_ = 95.78%. Consequently, subsample fusion can supply holistic information about the tomato plant and enhance DS expression to fulfill the accurate analysis of tomato plant DS. Manually distinguishing the leaves at different growth stages is a laborious and time-consuming task. The algorithms must be developed to distinguish leaves at different growth stages automatically by texture, color, and morphological information from an image.

#### 3.5.4 Effects of blue light on imaging

Besides, HSI systems using halogen lamps tend to have inferior SNR in the blue region (400–500 nm) of the electromagnetic spectrum, because a minimal amount of light is divided into the visible region for the HSI spectrometer with high spectral resolution (Mahlein et al., 2015). Previous works determined that low light intensity causes a dark current noise effect in the spectral profile of HSI images (Manea and Calin, 2015; Zhang et al., 2019). Moreover, the spectral curves in the 400–500 nm range, which depend on the amount of light absorbed by leaf pigments (chlorophyll, carotenoids, and anthocyanins), can reflect the physiological health information of plants (Zhao et al., 2016). DS affects metabolic reactions and the synthesis of photosynthetic pigments in the plant, and the response mechanism suggests that the visible light region is particularly sensitive to DS (Ihuoma and Madramootoo, 2019; Ihuoma and Madramootoo, 2020). High-frequency noise is suppressed by adding blue lights, and SNR is ameliorated effectively.

#### 3.5.5 Challenges, improvements, and developments

The detection of tomato plant DS still faces many challenges. For example, Susič et al. and Žibrat et al. indicated that both root-knot nematodes (biotic stress) and water deficiency (abiotic stress) lead to similar drought symptoms in plants (Susic et al., 2018; Zibrat et al., 2019). In the actual planting environment, the accurate differentiation of the biotic stress with similar symptoms of DS can contribute to the prevention of misidentification and inappropriate preventive measures affecting plant survival rate. Alordzinu et al. reported that plant responses to water stress are articulated by various physiological and biophysical changes and soil properties, they also assessed the water stress of tomato plants at different growth periods under different soils (Alordzinu et al., 2021). The mechanisms of resistance to tomato plant DS at different growth periods need to be further determined. Soils faced with widely cultivated tomato plants will be the focus of our future research. The specific effects of nutrients in the soil on the plant also need to be explored. Burnett et al. considered that spectroscopy can detect DS by investigating the potential biochemical changes before visual differences are observed, and metabolic responses to DS can be detected by HSI (Burnett et al., 2021). We should pay attention to the changes in the internal components of tomato leaves under DS, such as chlorophyll, soluble protein, catalase and so on. Moreover, it was shown that chlorophyll content, protein content decreased under DS, and the deeper the stress degree is, the lower the content is. The enzymatic activity of superoxide dismutase is significantly enhanced under severe DS, whereas catalase has a slight enhancement (Zgallai et al., 2006). We will attempt to estimate quantitatively the leaf metabolite concentrations pointing to tomato plant DS and achieve the early identification of DS. Two different types of leaves may be insufficient for estimating the physiological state of the plant system. The stress-induced changes in the physiological, biochemical, and molecular attributes of different plant organs need to be investigated. In this way, the health status, stress tolerance, and complex adaptation mechanisms of plant can be comprehensively assessed.

Additionally, some other aspects also need to be improved. (1) With the application of HSI in a greenhouse or a field, the uncontrollable lighting, complex background (soil, weeds, etc.), and mutual interference between plants can lead to incorrect DS analysis. Therefore, image correction and background segmentation should be considered. (2) In our work, the deep learning recognition models exhibited a barely prominent performance owing to the lack of training samples. Increasing the sampling data may improve the identification. Simultaneously, tomato plants with diverse stresses, including diseases and pests, various varieties, and different growth periods need to be further researched. Novel modeling algorithms deserve to be developed to accommodate heterogeneous samples and optimize classification performance. (3) Hyperspectral sensors can be mounted on the unmanned aerial vehicle (UAV) to perform large-scale DS detection and timely management. The trajectory, cycle, and height for the flight of the UAV, as well as the speed and range for the lens scanning of the HSI spectrometer, should be further explored.

During plant protection management, it is necessary to consider not only improving the accuracy of DS identification in plants, but also raising the adaptability of the plants to DS. Plant growth depends on the absorption of water from the soil and its transfer from roots to other plant parts. Therefore, understanding drought-induced changes to root anatomical traits is important to enhance plant drought adaptation (Alagoz et al., 2022). Different plant growth regulators respond differently to plant DS. Studying the effects of different plant growth regulators on plant physiological and biochemical processes can also help to promote the drought resistance of plants (Ghassemi et al., 2018). Along with the research on DS, the influence of fertilizer application on plants growth and biodiversity should also be discussed (Li et al., 2022). The use of chemical fertilizers remains controversial, so finding alternatives to chemical fertilizers for ecological sustainability is one of the pressing issues in modern agriculture (Sun et al., 2022). Overall, modern agricultural management aims to improve plant resistance while identifying stresses accurately and intervening scientifically.

## 4 Conclusion

In this work, the identification of tomato plant DS was performed using the multi-features of HSI and subsample fusion. The addition of blue lights removed the high-frequency noise in the 400–500 nm region. The reflectance spectra extracted from the HSI images showed that reflectance increased with the severity of tomato plant DS. Moreover, the image features extracted from the RIS by LeNet-5 positively affected the improvement of the model performance. Spectroscopy-image combination obtained good results with ACC_C_ = 94.09% and ACC_P_ = 91.57% for young leaves and ACC_C_ = 96.36% and ACC_P_ = 93.68% for mature leaves, which are superior to the identification accuracy values of the modeling by spectra or image features alone. Moreover, the classification accuracy of the subsample fusion model was further improved with ACC_C_ = 95.90% and ACC_P_ = 95.78%. In summary, the multi-features of HSI and the subsample fusion yielded an accurate identification of tomato plant DS under the supplementary illumination of blue lights. Applying HSI in complex environments, adding sample types and sample size, optimizing modeling algorithms, and utilizing of UAV equipped with an HSI spectrometer should be considered in future explorations to establish a stable, precise, and comprehensive classification model for various stress types and stress degrees.

## Data availability statement

The raw data supporting the conclusions of this article will be made available by the authors, without undue reservation.

## Author contributions

SW: Methodology, Conceptualization, Supervision, Funding acquisition. JM: Methodology, Validation, Formal analysis, Investigation, Visualization, Writing-original draft. WT: Writing–review & editing, Visualization. YT: Software, Investigation. MP: Investigation. ZZ: Validation, Formal analysis. LH: Resources, Methodology. LZ: Methodology, Supervision. JZ: Methodology, Validation. All authors contributed to the article and approved the submitted version. 
