# Supplementary material for: Drought stress identification of tomato plant using multi-features of hyperspectral imaging and subsample fusion
Source: Front Plant Sci. 2023 Feb 28;14:1073530. doi: 10.3389/fpls.2023.1073530 (PMC10011179; doi:10.3389/fpls.2023.1073530)
Supplement: Supplementary file 1 [file DataSheet_1.docx]

Drought stress identification of tomato plant using multi-features of hyperspectral imaging and subsample fusion

Shizhuang Weng ^1,*^, Junjie Ma ^1^, Wentao Tao ^1^, Yujian Tan ^1^, Meijing Pan ^1^, Zixi Zhang ^1^, Linsheng Huang ^1,*^, Ling Zheng ^1^, and Jinling Zhao ^1^

1. National Engineering Research Center for Agro-Ecological Big Data Analysis & Application, Anhui Univer-sity, 111 Jiulong Road Hefei, China; weng_1989@126.com (S.-Z.W.); junjie_P2020@163.com (J.-J.M.); linsheng0808@163.com (L.-S.H)

**Supporting Information**

**Hyperspectral imaging system** **supplemented with blue lights.**


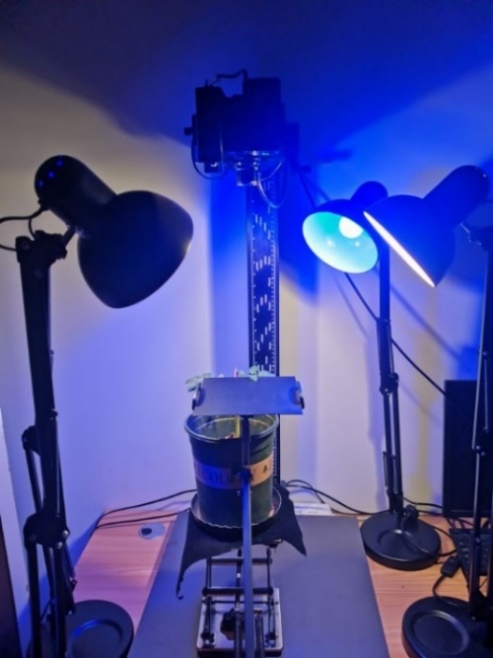


**Figure S1.** Hyperspectral imaging system for tomato leaf data acquisition in real environment.

**Selection of effective wavelengths (EWs).**

**Table S1.** The EWs selected by the genetic algorithm.

| Sample types | Number of Wavelengths | Wavelength(nm) |
| --- | --- | --- |
| Young leaves | 16 | 402, 475, 489, 515, 527, 535, 571, 573, 578, 602, 607, 624, 653, 687, 695, 711 |
| Mature leaves | 37 | 398, 433, 435, 502, 533, 562, 567, 582, 589, 591, 595, 611, 711, 713, 718, 740, 744, 755, 762, 773, 775, 791, 809, 811, 813, 822, 838, 842, 849, 871, 875, 878, 889, 898, 918, 967, 1000 |

**Parameter settings of image feature extraction.**

**Table S2.** Parameter settings of image feature extracted by LeNet-5.

| Method | Categories | Parameters |
| --- | --- | --- |
| LeNet | Young leaves | Conv_1:16,3×3,1, stride = (2,2), padding=1, BN (16), ReLU, Maxpool1d = (2,2);  Conv_2:16,3×3,1, stride = (2,2), padding=1, BN (16), ReLU, Maxpool1d = (2,2);  FC1:288, ReLU;  FC2:144, ReLU;  FC3:3;  Optimizer=’Adam’, loss=’ CrossEntropy’,  lr=0.00003, batch_size=8, num_epochs=120, weight_decay=0.8; |
|  | Mature leaves | Conv_1:16,3×3,1, stride = (2,2), padding=1, BN (16), ReLU, Maxpool1d = (2,2);  Conv_2:16,3×3,1, stride = (2,2), padding=1, BN (16), ReLU, Maxpool1d = (2,2);  FC1:288, ReLU;  FC2:144, ReLU;  FC3:3;  Optimizer=’Adam’, loss=’ CrossEntropy’,  lr=0.00002, batch_size=8, num_epochs=160, weight_decay=0.8; |

**The results of parallel experiment without blue light.**

**Table S3.** The difference in accuracy with blue light and without blue light.

| Method | Categories | Assembly ways | Accuracy (%) | |
| --- | --- | --- | --- | --- |
|  |  |  | ACCC | ACCP |
| SVM | Young leaves | Blue light | 90.90 | 87.36 |
|  |  | Without blue light | 93.50 | 86.00 |
|  | Mature leaves | Blue light | 94.09 | 91.57 |
|  |  | Without blue light | 91.06 | 88.88 |

**Parameter settings of models** **with full spectra and spectra of EWs.**

**Table S4.** Parameter settings of different classification models with full spectra and spectra of EWs.

| Data types | Categories | Methods | Parameters |
| --- | --- | --- | --- |
| Full spectra | Young leaves | SVM | C=369, kernel='poly', class_weight='balanced' |
|  |  | RF | n_estimators=17, random_state=0, max_depth=5, min_samples_leaf=6,  min_samples_split=30, max_features=16, criterion='entropy' |
|  |  | DenseNet | Conv_1:16,1×3,1, stride = (1,1), padding=1, ReLU;  Conv_2:16,1×3,1, stride = (1,1), padding=1, ReLU;  Conv_3:32,1×3,1, stride = (1,1), padding=1, ReLU, Maxpool1d = (2,2);  FC1:128, ReLU;  FC2:64, ReLU;  FC3:3;  Optimizer=’ RMSprop’, loss=’ CrossEntropy’,  lr=0.000058, batch_size=9, num_epochs=500, weight_decay=0.035; |
|  | Mature leaves | SVM | C=530, kernel='poly' |
|  |  | RF | n_estimators=5, random_state=0, max_depth=8, min_samples_leaf=5, min_samples_split=13, max_features=39 |
|  |  | DenseNet | Conv_1:16,1×3,1, stride = (1,1), padding=1, ReLU;  Conv_2:16,1×3,1, stride = (1,1), padding=1, ReLU;  Conv_3:32,1×3,1, stride = (1,1), padding=1, ReLU, Maxpool1d = (2,2);  FC1:128, ReLU;  FC2:64, ReLU;  FC3:3, ReLU;  Optimizer=’ RMSprop’, loss=’ CrossEntropy’,  lr=0.000061, batch_size=10, num_epochs=500; |
| Spectra of EWs | Young leaves | SVM | C=156, kernel='rbf' |
|  |  | RF | n_estimators=3, random_state=0, criterion='entropy', max_depth=5,  min_samples_leaf=3, min_samples_split=4, max_features=15 |
|  |  | DenseNet | Conv_1:16,1×3,1, stride = (1,1), padding=1, ReLU;  Conv_2:16,1×3,1, stride = (1,1), padding=1, ReLU;  Conv_3:32,1×3,1, stride = (1,1), padding=1, ReLU, Maxpool1d = (2,2);  FC1:128, ReLU;  FC2:64, ReLU;  FC3:3;  Optimizer=’ RMSprop’, loss=’ CrossEntropy’,  lr=0.000088, batch_size=14, num_epochs=500, weight_decay=0.0049; |
|  | Mature leaves | SVM | C=400, kernel='poly' |
|  |  | RF | n_estimators =11, random_state = 0, max_depth = 6, min_samples_leaf = 3, min_samples_split = 2, max_features = 27 |
|  |  | DenseNet | Conv_1:16,1×3,1, stride = (1,1), padding=1, ReLU;  Conv_2:16,1×3,1, stride = (1,1), padding=1, ReLU;  Conv_3:32,1×3,1, stride = (1,1), padding=1, ReLU, Maxpool1d = (2,2);  FC1:128, ReLU;  FC2:64, ReLU;  FC3:3;  Optimizer=’ RMSprop’, loss=’ CrossEntropy’,  lr=0.0009, batch_size=10, num_epochs=500, weight_decay=0.0155; |

SVM:

C—Penalty parameter C of the error term.

kernel—Specifies the kernel type to be used in the algorithm.

class_weight—Adjust the weight ratio to prevent data with small samples from being ignored

RF:

n_estimators—The number of decision trees in a random forest.

random_state—Controls both the randomness of the bootstrapping of the samples used when building trees.

criterion—The function to measure the quality of a split.

max_features—Maximum feature number of random forest partition.

max_depth—The maximum depth of the decision tree.

min_samples_split—The minimum number of samples required to split an internal node.

min_samples_leaf—The minimum number of samples required to be at a leaf node.

DenseNet:

lr—Make the objective function converge to the local minimum in a suitable time.

batch_size—The number of training samples sent into the network for each training.

num_epochs—Total number of training sessions for all samples.

weight_decay—The model overfitting can be reduced to some extent.

**The reflectance image set (RIS) was determined by superposing one by one for the selected reflectance images.**

**Table S5.** Determination of RIS.

| Method | Categories | Different combinations | Accuracy (%) | |
| --- | --- | --- | --- | --- |
|  |  |  | ACC_C_ | ACC_P_ |
| SVM | Young leaves | 695nm | 94.54 | 86.31 |
|  |  | 695nm and 711nm | 94.54 | 90.52 |
|  |  | 695nm,711nm and 607nm | 94.09 | 91.57 |
|  |  | 695nm,711nm,607nm and 687nm | 95.90 | 90.52 |
|  |  | 695nm,711nm,607nm,687nm and 653nm | 93.63 | 84.21 |
|  | Mature leaves | 567nm | 92.27 | 91.57 |
|  |  | 567nm and 773nm | 95.90 | 92.63 |
|  |  | 567nm,773nm and 791nm | 95.45 | 92.63 |
|  |  | 567nm,773nm,791nm and 822nm | 96.36 | 93.68 |
|  |  | 567nm,773nm,791nm, 822nm and 762nm | 96.81 | 92.63 |

**Parameter settings of models with spectroscopy-image combination.**

**Table S6.** Parameter settings of models with spectroscopy-image combination.

| Categories | Methods | Parameters |
| --- | --- | --- |
| Young leaves | SVM | C=532 |
|  | RF | n_estimators=4, random_state=0, max_depth=4, min_samples_leaf=2, min_samples_split=2, max_features=3 |
|  | DenseNet | Conv_1:16,1×3,1, stride = (1,1), padding=1, ReLU;  Conv_2:16,1×3,1, stride = (1,1), padding=1, ReLU;  Conv_3:32,1×3,1, stride = (1,1), padding=1, ReLU, Maxpool1d = (2,2);  FC1:128, ReLU;  FC2:64, ReLU;  FC3:3;  Optimizer=’ RMSprop’, loss=’ CrossEntropy’,  lr=0.00009, batch_size=14, num_epochs=500, weight_decay=0.018; |
| Mature leaves | SVM | C=600, kernel='poly' |
|  | RF | n_estimators =26, criterion='entropy', random_state = 0, max_depth = 7, min_samples_leaf = 1, min_samples_split = 2, max_features = 11 |
|  | DenseNet | Conv_1:16,1×3,1, stride = (1,1), padding=1, ReLU;  Conv_2:16,1×3,1, stride = (1,1), padding=1, ReLU;  Conv_3:32,1×3,1, stride = (1,1), padding=1, ReLU, Maxpool1d = (2,2);  FC1:128, ReLU;  FC2:64, ReLU;  FC3:3;  Optimizer=’ RMSprop’, loss=’ CrossEntropy’,  lr=0.00016, batch_size=10, num_epochs=500, weight_decay=0.0036; |

**Parameter settings of models with subsample fusion.**

**Table S7.** Parameter settings of models with subsample fusion.

| Methods | Parameters |
| --- | --- |
| SVM | C=717, kernel='poly', class_weight='balanced' |
| RF | n_estimators=27, random_state=0, max_depth=10, min_samples_leaf=3, min_samples_split=2, max_features=27 |
| DenseNet | Conv_1:16,1×3,1, stride = (1,1), padding=1, ReLU;  Conv_2:16,1×3,1, stride = (1,1), padding=1, ReLU;  Conv_3:32,1×3,1, stride = (1,1), padding=1, ReLU, Maxpool1d = (2,2);  FC1:128, ReLU;  FC2:64, ReLU;  FC3:3;  Optimizer=’ RMSprop’, loss=’ CrossEntropy’,  lr=0.00007, batch_size=10, num_epochs=500; |

**Classification results using images alone.**

**Table S8.** The classification accuracy with using images alone.

| method | Sample types | Accuracy (%) | |
| --- | --- | --- | --- |
|  |  | ACC_C_ | ACC_P_ |
| LeNet-5 | Young leaves | 0.6682 | 0.6316 |
|  | Mature leaves | 0.7182 | 0.6737 |
